# Identification of Key Genes and miRNAs Affecting Osteosarcoma Based on Bioinformatics

**DOI:** 10.1155/2022/1015593

**Published:** 2022-11-16

**Authors:** Le Li, Xin Zhou, Wencan Zhang, Ran Zhao

**Affiliations:** ^1^Department of Orthopedic Surgery, Qilu Hospital of Shandong University, Jinan, Shandong Province, China; ^2^Department of Burns and Plastic Surgery, Shandong Provincial Hospital Affiliated to Shandong First Medical University, Jinan, Shandong Province, China

## Abstract

**Methods:**

GSE70367 and GSE69470 were obtained from the GEO database. The differentially expressed genes (DEGs) and miRNAs were analyzed using the GEO2R tool and then visualized with R software. Moreover, the targets of the miRNAs in the DEGs were screened and then used for enrichment analysis. Besides, the STRING database and Cytoscape were applied to illustrate the protein-protein interaction network. RT-qPCR was performed to measure the expression of key genes and miRNAs. Western blot was applied to detect the signaling pathway.

**Results:**

9 upregulated genes and 39 downregulated genes in GSE69470 were identified as the DEGs, and 31 upregulated genes and 56 downregulated genes in GSE70367 were identified as the DEGs. Moreover, 21 common genes were found in the DEGs of GSE70367 and GSE69470. The enrichment analysis showed that the common DEGs of GSE70367 and GSE69470 were related with cell development, covalent chromatin modification, and histone modification and involve in the regulation of MAPK, mTOR, and AMPK pathways. Besides, the miRNAs including miR-543, miR-495-3p, miR-433-3p, miR-381-3p, miR-301a-3p, miR-199b-5p, and miR-125b-5p were identified as the biomarkers of osteosarcoma. In addition, the target genes including HSPA5, PPARG, MAPK14, RAB11A, RAB5A, MAPK8, LEF1, HIF1A, CAV1, GS3KB, FOXO3, IGF1, and NFKBIA were identified as hub nodes. It was found that miR-301a-3p expression was decreased and mRNA expression of RAB5A and NFKBIA was increased in the pathological tissues. The AKT-PI3K-mTOR signaling pathway was activated in pathological tissues.

**Conclusion:**

In this study, 7 miRNAs and 13 hub genes were identified, which might be candidate markers. miR-301a-3p, RAB5A, and NFKBIA were abnormally expressed in osteosarcoma tissues.

## 1. Introduction

Osteosarcoma is a frequent malignant bone disease in children and older patients, which is characterized with poor prognosis including physical disability and metastases [1, 2]. Surgery excision, radiotherapy, and chemotherapy have been widely used for osteosarcoma treatment, which can effectively inhibit the development of the tumor progression in the early stage [3]. Nevertheless, considerable patients have been confirmed to be at the advanced stage in their first clinical diagnosis. Moreover, high metastasis rates of osteosarcoma also make the clinical intervention become tricky and then lead to treatment failure [4]. Although the survival times of the patients have been significantly prolonged with the modern medicine techniques, the treatment effect remains unsatisfactory for patients [5, 6]. At present, some reports have focused on revealing the potential mechanism of osteosarcoma, which can provide valuable reference for the progression of medicine strategies [7, 8]. At present, there have been many studies on the molecular mechanism of osteosarcoma, and several osteosarcoma-driving genes have been identified, such as TP53, RB1, and PTEN. There have also been targeted drugs for osteosarcoma, such as pazopanib, appatinib, cabotinib, and ivermex. However, these studies have not clearly explained the pathogenesis and metastasis of osteosarcoma. Therefore, it is urgent to further study the potential molecular mechanism of osteosarcoma cells, identify reliable molecular markers, and identify new drug targets.

Microarray analysis is a useful method which has been used for screening the key genes in diseases [9]. Recently, the academic and guiding value of bioinformatics methods on improving the clinical practice have been proven by numerous researches [10]. MicroRNA is a class of the short noncoding RNA with 18-20 nucleotides, which plays a great part in the cellular life activity [11]. The abnormal expression of miRNA is a biomarker event in multiple diseases, especially in caner. In osteosarcoma, many studies have indicated that miRNA can regulate the cellular phenotype to influence the progression of the tumor via intervening the expression of key proteins [12]. However, the miRNA-mRNA interaction network of osteosarcoma is still far from complete clarification.

In this project, the purpose was to identify the pivotal biomarkers and related mechanism of the osteosarcoma using bioinformatics methods through obtaining the open-source datasets in the GEO database.

## 2. Materials and Methods

### 2.1. Data Source

We searched the datasets comparing mRNA or miRNA expression profiles of osteosarcoma and normal samples using “osteosarcoma” as search terms for the GEO datasets (https://www.ncbi.nlm.nih.gov/geo/). The datasets including GSE70367 and GSE69470 were obtained. GSE69470 contained the expression profile of 15 samples, including 10 osteosarcoma samples and 5 normal samples, which was based on platform GPL20275. For GSE70367 based on GPL16384, 5 samples of tumor cell lines and 1 sample of the hMSC cell line were used for analysis.

### 2.2. Identification of Differentially Expressed Genes

The DEGs of the datasets were analyzed with the GEO2R tool of the GEO database to obtain the related matrix files. The genes with the |logFC| > 2 and *P* value < 0.05 were selected as the DEGs.

### 2.3. KEGG and GO Enrichment Analysis

The targets of the DEGs were predicted with the mirDIP database (http://ophid.utoronto.ca/mirDIP/index.jsp), and the top 5% genes in the results were selected as potential targets of the DEGs in GSE70367 and GSE69470. The KEGG and GO enrichment of DEGs was performed by the DAVID database. In brief, the targets of the DEGS were uploaded into the DAVID database. The pathways and the related functional modules in the results with *P* value < 0.05 were visualized with the R language.

### 2.4. Network Analysis

The protein-protein interaction network was performed to identify the hub nodes of the DEGs. Briefly, the targets of the DEGs were uploaded to the STRING database (https://cn.string-db.org/) to analyze and obtain protein interaction information, and then, Cytoscape software was applied to visualize the PPI network.

### 2.5. Clinical Tissues

The pathological tissues and adjacent healthy tissues were requested from the Qilu Hospital of Shandong University. The experiments were approved by the ethics committee of the hospital. Besides, all tissues were frozen at -70°C.

### 2.6. qRT-PCR

The RNAs in the tissues were extracted with a TRIzol reagent. The commercial kit (Shanghai Lianmai Biological Engineering Co., Ltd., Shanghai, China) was applied for the reverse transcription of cDNA. Subsequently, the PCR reaction was performed for the quantification of the genes. Moreover, the abundance of the RNAs were measured with the 2^−*ΔΔ*Ct^ method.

### 2.7. Western Blot Analysis

The protein was extracted by the RIPA buffer. Protein was separated by SDS-PAGE and transferred to the nitrocellulose membrane and blocked in 5% skim milk powder solution for 2 h. Then, the membrane was incubated with a primary antibody at 4°C for 12 h. Then, the membrane was incubated by a second antibody at 25°C for 2 h. The protein bands were colored, and the gray value was read under ImageJ software.

### 2.8. Immunohistochemistry (IHC)

IHC was conducted using paraffin-embedded tissue sections. After being deparaffinized and hydrated, the antigen was extracted at 95°C. After treating with 3% H_2_O_2_, sections were incubated with primary antibody at 4°C overnight and then treated with a second antibody at 37°C for 30 min. Staining was conducted using DAB (Golden Bridge, China).

### 2.9. Data Analysis

The experiments were repeated for three times, independently. SPSS 19.0 and GraphPad Prism 8.0 was applied for data analysis and visualization, respectively. Moreover, the chi-squared test or ANOVA with Tukey's post hoc test was selected for calculating the difference of data, and *P* < 0.05 represented that the difference was statistically significant.

## 3. Results

### 3.1. DEG Identification

**Figure 1:**
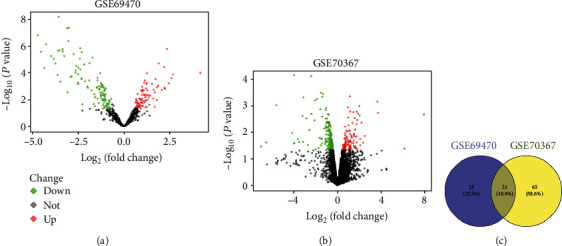
The DEGs in GSE69470 and GSE70367 were visualized with volcano plots. (a) The DEGs in GSE69470. (b) The DEGs in GSE70367. (c) The common genes of GSE69470 and GSE70367 were screened by Venn diagram.

**Figure 2:**
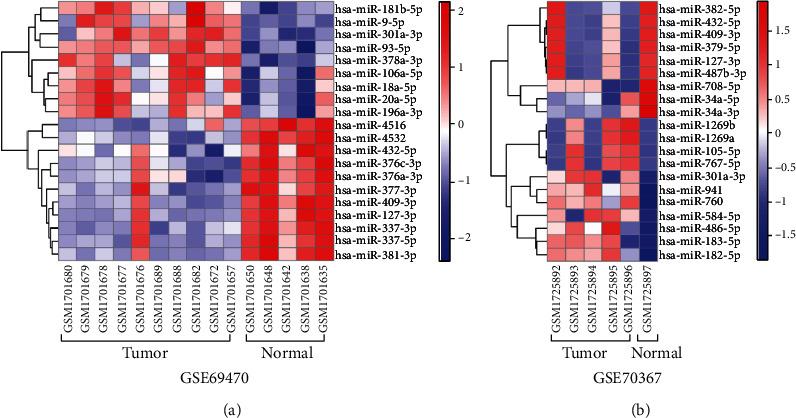
The expressions of DEGs in the samples of GSE69470 and GSE70367 were visualized by heat map: (a) the DEGs in GSE69470; (b) the DEGs in GSE70367.

To investigate the gene profiles of OS, GSE70367 and GSE69470 were obtained from the GEO database and then analyzed with the GEO2R tool. 9 upregulated DEGs and 37 downregulated DEGs were found in GSE69470, and 31 upregulated DEGs and 55 downregulated DEGs were found in GSE70367 (Figures 1(a) and 1(b)). Moreover, the abundance of the DEGs of GSE70367 and GSE69470 were exhibited in Figure 2. Moreover, 21 downregulated genes were found in GSE70367 and GSE69470 (Figure 1(c)). Those observations suggested that there were significant differences in gene profile of tumor cells and normal cells.

### 3.2. Identification of Function Model

**Figure 3:**
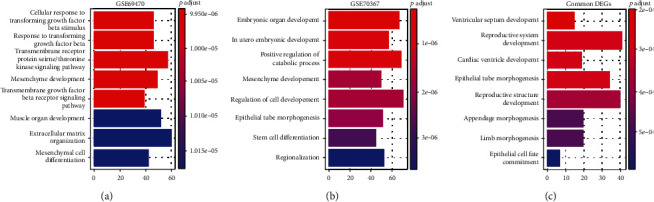
GO enrichment analysis of the DEGs. (a) The GO enrichment analysis of the DEGs in GSE69470. (b) The GO enrichment analysis of the DEGs in GSE70367. (c) The GO enrichment analysis of the common genes in GSE69470 and GSE70367.

To investigate the functions of the genes in the progression of osteosarcoma, the targets of the DEGs in GSE70367 and GSE69470 were analyzed with GO enrichment. The results proved that the DEGs in GSE69470 were associated with the regulation of extracellular structure, regulation of protein serine/threonine kinase activity, regulation of GTPase activity, and so on. For GSE70367, the DEGs were related with regulation of cell development, positive regulation of catabolic process, skeletal system development, and so on (Figures 3(a) and 3(b)). Moreover, the common DEGs of GSE70367 and GSE69470 were also related with regulation of cell development, covalent chromatin modification, and histone modification (Figure 3).

### 3.3. KEGG Enrichment Analysis

**Figure 4:**
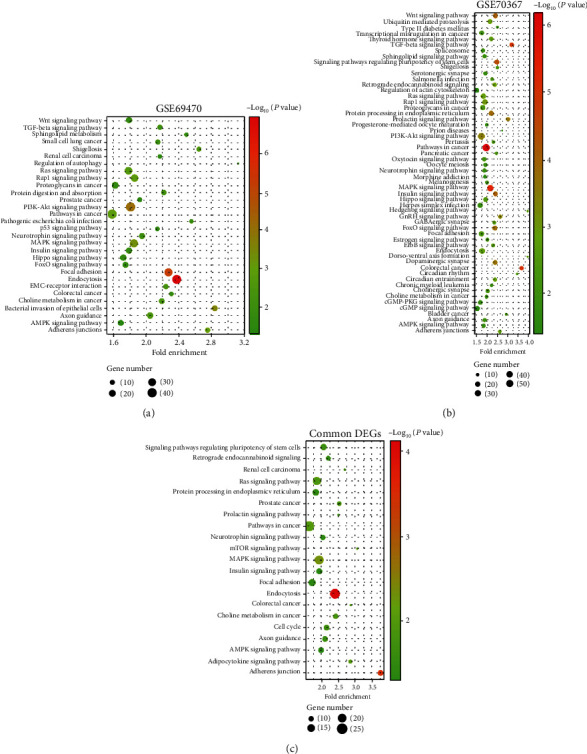
The KEGG enrichment analysis of the DEGs. (a) The KEGG enrichment analysis of the DEGs in GSE69470. (b) The KEGG enrichment analysis of the DEGs in GSE70367. (c) The KEGG enrichment analysis of the common genes in GSE69470 and GSE70367.

For revealing the regulation mechanisms of osteosarcoma, the DEGs of the datasets were analyzed with KEGG enrichment. It was proven that the DEGs in GSE69470 were connected with the extracellular matrix (ECM) receptor interaction, focal adhesion, PI3K/AKT pathways, P53 pathways, TGF-*β*, Wnt pathway, etc. (Figure 4(a)). The DEGs in GSE70367 were related with the ECM-receptor interaction, PI3K/AKT pathways, TGF-*β* pathway, Hippo pathway, p53 pathway, Wnt pathway, and so on (Figure 4(b)). In addition, the common DEGs of GSE69470 and GSE70367 were related with the MAPK signaling pathway, mTOR signaling pathway, AMPK signaling pathway, Ras signaling pathway, and so on (Figure 4(c)).

### 3.4. PPI Network

**Figure 5:**
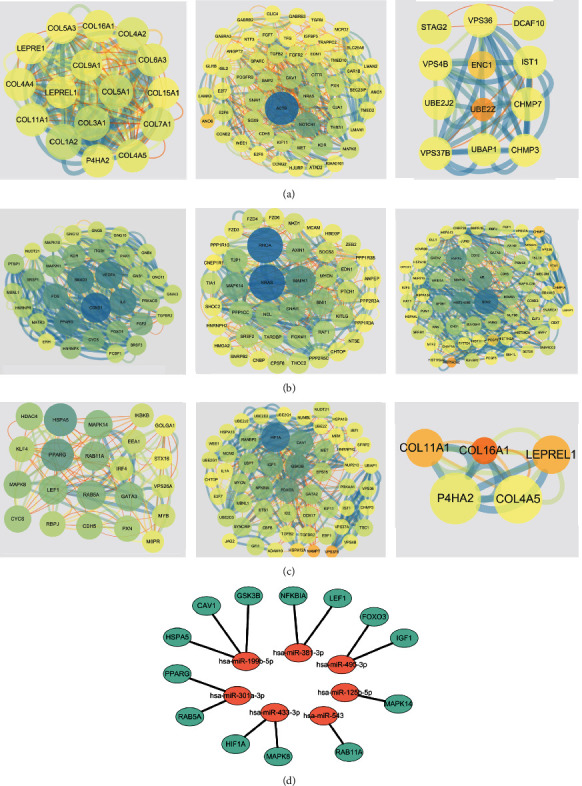
PPI-network analysis and miRNA-mRNA network analysis of DEGs and the related targets. (a) The DEGs in GSE69470 (big and blue sizes were selected as hub nodes). (b) The DEGs in GSE70367 (big and blue sizes were selected as hub nodes). (c) The common genes of GSE69470 and GSE70367. (d) miRNA-mRNA network (red: hsa-miR-433-3p; green: protein).

**Figure 6:**
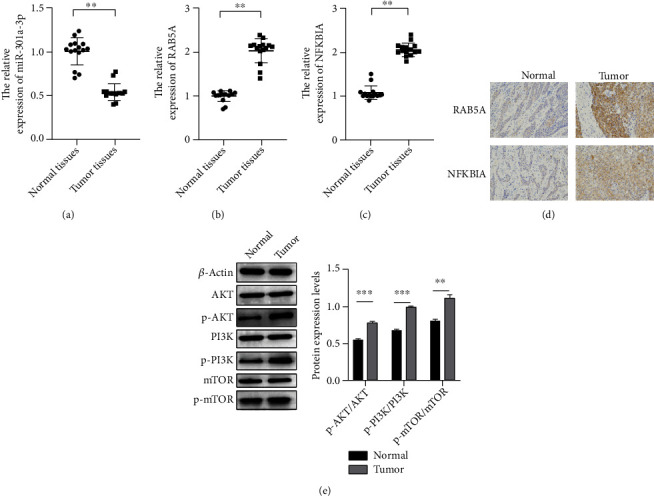
Decreased miR-301a-3p and increased RAB5A and NFKBIA were detected in the pathological tissues. (a–c) The related abundance of miR-301a-3p (a), RAB5A (b), and NFKBIA (c) in the pathological tissues. (d) IHC staining for RAB5A and NFKBIA was performed. (e) Protein expression was measured via western blot.

To illustrate the molecular mechanism of osteosarcoma, the protein interactions of DEGs were analyzed to obtain the hub nodes. The results mirrored that for GSE69470, 3 clusters were found in the targets, including cluster 1 with 16 nodes and 234 edges, cluster 2 with 54 nodes and 520 edges, and 84 nodes and 480 edges (Figure 5(a)). For GSE70367, 3 clusters were found in targets, including cluster 1 with 33 nodes and 322 edges, cluster 2 with 43 nodes and 298 edges, and cluster 3 with 71 nodes and 376 edges (Figure 5(b)). Moreover, for the common miRNAs of GSE69470 and GSE70367, there were three clusters including cluster 1 with 22 nodes and 126 edges, cluster 2 with 56 nodes and 308 edges, and cluster 3 with 5 nodes and 20 edges. The results showed that the factors including HSPA5, PPARG, MAPK14, RAB11A, RAB5A, MAPK8, LEF1, GATA3, HIF1A, CAV1, GS3KB, FOXO3, IGF1, and NFKBIA were selected as the hub nodes (Figure 5(c)). In addition, the miRNA-mRNA network was also established (Figure 5(d)). Besides, to verify the relationship of the genes and the progression of osteosarcoma, the screened genes were identified with the published studies or qRT-PCR. It was found that decreased miR-301a-3p and increased RAB5A and NFKBIA were detected in the pathological tissues (Figures 6(a)–6(c)). In addition, IHC results showed that RAB5A and NFKBIA were highly expressed in pathological tissues (Figure 6(d)). The AKT-PI3K-mTOR signaling pathway was activated in pathological tissues (Figure 6(e)).

## 4. Discussion

Osteosarcoma is one of the dangerous diseases with high incidence, and there are few effective strategies to completely heal this disease [5]. Bioinformatics analysis has been verified as a promising strategy for identifying the biomarkers and researching the molecular mechanism of cancer [13]. In this investigation, the datasets including GSE69470 and GSE70367 were obtained from the GEO database and then used for identifying the hub nodes in osteosarcoma.

Osteosarcoma is characterized with aberrant expression of genes which may involve the some malignant behaviors of the tumor cells. In this project, the expressions of genes in tumor cell lines and normal cell lines were investigated, and 21 downregulated genes were found in GSE69470 and GSE70367. Moreover, downregulation of miR-127-3p, miR-154-5p, miR-323a-3p, miR-409-3p, miR-431-5p, miR-432-5p, miR-433-3p, miR-485-3p, miR-487b-3p, miR-495-3p, and miR-125b-5p was related with cancer development. For instance, miR-127-3p serves as an inhibitor role in the progression of multiple tumors such as glioblastoma and prostate cancer [14, 15]. For osteosarcoma, all of those miRNAs are also involved in the malignant behaviors such as invasion and proliferation.

Cancer development always involves the changes of multiple signaling pathways, such as PI3K/AKT pathways, P53 pathway, and Wnt/*β*-catenin pathway [16, 17]. For osteosarcoma, the disorder of the cellular signal pathways has also been confirmed as the direct reasons leading to tumor cell proliferation and invasion [18]. The PI3K/AKT pathway is related with cellular proliferation, and the activated PI3K/AKT pathway has been confirmed to involve the progression of multiple cancers. The study of Yang et al. has indicated that the PI3K/AKT pathway was aberrantly activated in the osteosarcoma cells, and inhibiting the PI3K/AKT pathway could effectively impede the proliferation of tumor cells [19]. In this project, it was proven that the DEGs in GSE69470 or GSE70367 were associated with multiple pathways including the PI3K/AKT, TGF-*β*, Hippo, P53, Wnt, and MAPK pathways. The dysfunctions of signal pathways in tumor cells are closely connected with the miRNA disorder. Increased miR-127-3p, miR-495-3p, and miR-125b-5p have been proven to take part in suppressing the activity of the PI3K/AKT pathway [20–22]. Moreover, the report has proven that miR-409-3p involves regulation of MAPK to block cervical cancer development [23]. Beside, decreased miR-301a-3p was also found in the pathological tissues.

miRNA can obstruct the translation progression of proteins via inducing the degradation of the special mRNAs [23]. In this project, the targets of the DEGs in GSE69470 and GSE70367 were predicted and used to reveal the molecular mechanism of osteosarcoma. It was found that the factors including HSPA5, PPARG, MAPK14, RAB11A, RAB5A, MAPK8, LEF1, GATA3, HIF1A, CAV1, GS3KB, FOXO3, IGF1, NFKBIA, and so on were selected as the hub nodes. The study has indicated that HSPA5 inhibition is a promising method for inducing the endoplasmic reticulum stress, autophagy, and apoptosis of tumor cells [24]. MAPK family serves as an important role in the progression of multiple tumors. The studies have indicated that increased MAPK8 plays a critical role in the development of colorectal cancer, and MAPK14 downregulation could effectively impede the poor behaviors of the clear cell renal cell carcinoma [25, 26]. Increasing studies have revealed that the disorder of the RAS oncogene family was related with the progression of tumor. In this study, RAB11A and RAB5A were also identified as the hub nodes of osteosarcoma. RAB11A involves the regulation of the Wnt/*β*-catenin pathway to promote the deterioration of prostate cancer, and RAB5A upregulation is related with the proliferation invasion and EMT of ovarian cancer [15, 27]. LEF1 upregulation is related to the resistance of cancer. Fakhr et al. have proven that LEF1 silence could improve the lethal effect of chemotherapy drugs on colorectal cancer cells [28]. HIF1A serves as a key role in regulating the formation of the blood vessel under the hypoxic condition. Some reports have indicated that HIF1A upregulation is related with the invasion and metastasis of tumor cells. In this study, HIF1A was also identified as a hub node. Moreover, CAV1, GS3KB, FOXO3, IGF1, and NFKBIA have also been proven as the biomarkers for prognosis of multiple cancers. Moreover, increased RAB5A and NFKBIA were detected in the pathological tissues.

In conclusion, in this study, 7 miRNAs and 13 hub genes were identified, which might be candidate markers. miR-301a-3p, RAB5A, and NFKBIA were abnormally expressed in osteosarcoma tissues. However, one of the limitations of this study was that no more experiments have been conducted to verify whether miR-301a-3p, RAB5A, and NFKBIA affect tumor progression. In addition, this study lacks more dataset analysis to verify the conclusions of this study.

## Data Availability

The data used to support the findings of this study are included within the article.
